# I Mind My Own Business and Expect Others to Do the Same: Conversations About Declining Financial Capacity

**DOI:** 10.1093/geroni/igab046.1255

**Published:** 2021-12-17

**Authors:** Mingyang Zheng, Marguerite DeLiema

**Affiliations:** 1 Radford University, Minneapolis, Minnesota, United States; 2 University of Minnesota, Twin Cities, SAINT PAUL, Minnesota, United States

## Abstract

Introduction: One of the smartest ways to prepare for declines in financial decision making capacity is to appoint an agent under power of attorney for finances and to share important financial information and preferences with trusted family or friends. Yet only 12% of older Americans with children think that they’ll need help with their finances as they age, and more than half are uncomfortable talking about their finances with children. Method: We conducted four in-depth interviews with older adults and four focus groups with Black, Latino, low income, and low-middle income adults aged 65 and older. An average of 9 participants were in each 2-hour focus group. Results: Barriers included lack of awareness, denial of future changes in capacity, lack of trustworthy surrogate decision-makers, shame about one’s financial situation, desire for privacy, fear of being a burden, and resistance to overtures by children. Barriers differed by ethnicity and socioeconomic status, with lower income older adults having less knowledge of advance planning and Powers of Attorney. Implications: Significant education is needed around Powers of Attorney and how to begin the advance planning process. Study findings informed the Thinking Ahead Roadmap, a guide to facilitate planning and communication around future money management. The Roadmap uses an empowerment framework to motivate individuals to appoint trusted financial advocates and prepare them for a smooth transition in money management, thereby reducing risk of exploitation, costly mistakes, and family conflict.

